# Quantitative Evaluation of Blending Behavior between Virgin Asphalt and Aged Asphalt Incorporating a New Bio-Based Warm-Mix Rejuvenator

**DOI:** 10.3390/ma17164061

**Published:** 2024-08-15

**Authors:** Le Yu, Shiyuan You, Zhaoyi He, Dingbang Wei, Lin Kong

**Affiliations:** 1 School of Civil Engineering, Chongqing Jiaotong University, Chongqing 400074, China; 611200111015@mails.cqjtu.edu.cn (L.Y.); ysyyola@163.com (S.Y.); 2 Chongqing City Construction Investment Group Co., Ltd., Chongqing 400023, China; 3 National and Local Joint Engineering Laboratory of Traffic Civil Engineering Materials, Chongqing Jiaotong University, Chongqing 400074, China; 4 Gansu Transport Planning, Survey and Design Institute Co., Ltd., Lanzhou 730030, China; weidingbang@163.com; 5 Gansu Province Highway Traffic Construction Group Co., Ltd., Lanzhou 730030, China; 6 School of Civil Engineering, Southwest Jiaotong University, Chengdu 610031, China; konglin@my.swjtu.edu.cn

**Keywords:** virgin asphalt, aged asphalt, recycled asphalt, bio-based warm-mix rejuvenator, regenerative blending degree, quantitative evaluation

## Abstract

The blending degree between virgin asphalt and aged asphalt has a significant effect on road performance of reclaimed asphalt mixture. This study presented an innovative examination of blending behavior between virgin asphalt and aged asphalt incorporating a new bio-based warm-mix rejuvenator (BWR) by utilizing Atomic Force Microscopy (AFM). Through analyzing the variation of several micro-morphology parameters between virgin asphalt and aged asphalt (or recycled asphalt) after blending, an index of regenerative blending degree (RBD) was proposed to quantitatively evaluate their blending behavior, and the effect of various blending temperatures and durations on regenerative blending degree was investigated. The results show that the regenerative blending degree between virgin asphalt and aged asphalt was higher than that between virgin asphalt and recycled asphalt under the same blending condition. A clear linear correlation was observed between the regenerative blending degree calculated by 3D micro-morphology parameters and the dosage of bio-based warm-mix rejuvenator in recycled asphalt, with a correlation coefficient of 0.98. With the increase in blending duration, the regenerative blending degree between virgin asphalt and recycled asphalt increased first and then decreased, but continued to improve with the increase in blending temperature, which indicates that a higher blending temperature and prolonging the blending duration properly have a positive effect on the blending processing between virgin asphalt and recycled asphalt. Compared with the regenerative blending degree calculated by 2D micro-morphology parameters, the regenerative blending degree calculated by 3D micro-morphology parameters is more reasonable to quantify the blending behavior between virgin asphalt and recycled asphalt.

## 1. Introduction

Nearly 80 million cubic meters of reclaimed asphalt pavement (RAP) are produced in the maintenance engineering of asphalt pavement every year in China, but the content of RAP in recycled asphalt mixture is usually less than 30%, which is inconsistent with the expectations of researchers [1,2]. How to reduce the risk of environmental pollution and minimize the engineering cost by the high-content and high-quality utilization of RAP has been a hot spot in the field of road engineering [3,4]. Hot recycling technology is considered to be one of the effective ways to consume vast amounts of RAP and can blend aged asphalt with virgin asphalt (or rejuvenators) at high temperatures to restore its physicochemical properties, which means that the blending degree of virgin asphalt and aged asphalt has a great effect on improving road performance and increasing RAP content in recycled asphalt mixture [5,6,7].

Previous studies have analyzed the blending phenomenon by various macro and micro tests and found that partial blending is more in line with the actual blending state of virgin asphalt and aged asphalt compared with no blending and total blending [8,9,10]. To distinguish virgin asphalt and aged asphalt (or recycled asphalt) and accurately quantify the blending phenomenon, some researchers have used staged-extraction method to assess the blending degree by comparing the properties variation of virgin asphalt and aged asphalt after blending [11,12]. Meanwhile, the tracer method can characterize the blending process by adding tracer into asphalt [7,10]. Rinaldini has added titanium dioxide tracer into virgin asphalt, and the distribution of titanium dioxide in the recycled asphalt mixture, which was determined by energy-dispersive X-ray spectroscope, was used to evaluate the blending phenomenon [13]. Due to the differences in molecular size and functional group types between aged asphalt and virgin asphalt, Gel permeation chromatography (GPC) and Fourier transform infrared spectroscopy (FTIR) were used as indirect methods to reflect the blending degree by the variation of large molecular size and sulfoxide group (or carbonyl group), respectively [14,15]. Subsequently, more direct methods such as Computed tomography (CT), Laser scanning confocal microscope (LSCM), and Atomic force microscopy (AFM) were conducted to realistically characterize the blending state of virgin asphalt and aged asphalt [13,16,17]. Nahar used Atomic force microscopy to provide a first observation of blending-zone and transition-zone between RAP binder and virgin asphalt and explore their interaction and blending degree [18]. Zou proposed a three-dimensional fluorescence image method to quantitatively evaluate the blending degree by using a laser scanning confocal microscope and SBS tracer, and the effect of preheating duration and temperature on the blending process were investigated [16,19]. The above research shows that the three-dimensional characterization of the blending phenomenon between virgin asphalt and aged asphalt has been a popular research method in recent years.

To further improve the road performance of aged asphalt in an environment-friendly way, bio-based warm-mix rejuvenators have been successively used to supply the light components lost by oxidation or volatilization and restore its colloidal structure [20,21]. However, some studies have pointed out that the oxygen content and microstructure of bio-oil are significantly different from asphalt [22,23], so aged asphalt incorporated with bio-based warm-mix rejuvenators has a different colloidal structure compared with virgin asphalt [24], which may lead to unknown impact on the blending degree between virgin asphalt and recycled asphalt. Blending temperature and duration will also affect the blending process of virgin asphalt and aged asphalt [25], but there were no relevant reports on the variation of blending degree under different blending conditions (temperatures and durations) after adding bio-based warm-mix rejuvenators into aged asphalt. Furthermore, with the rapid development of computer technology, researchers have used molecular dynamics (MD) simulations to study the effects of rejuvenators with different molecular structures on the blending and diffusing process of virgin asphalt and aged asphalt under various blending conditions and found that the blending behavior of rejuvenators in aged asphalt followed the Fickian law and a lower dosage of rejuvenators had a better blending degree [26,27,28].

The purpose of this study is to present an innovative quantitative method of blending phenomenon between virgin asphalt and aged asphalt by utilizing Atomic Force Microscopy (AFM) incorporating a new bio-based warm-mix rejuvenator (BWR) which was self-developed by the research group. According to the variation of several 2D and 3D micro-morphological parameters between virgin asphalt and aged asphalt (or recycled asphalt) after blending, the regenerative blending degree (RBD) was proposed to quantitatively evaluate their blending degree, and the effect of various blending temperatures and durations on regenerative blending degree was investigated.

## 2. Materials and Methods

### 2.1. Materials

#### 2.1.1. Asphalt Binders

Virgin asphalt (VA) was Zhonghai 70# asphalt provided by Chongqing Zhongjiao Renewable Resources Development Co., Ltd. (Chongqing, China), which is a local company in Chongqing, China. Aged asphalt (AA) could be prepared based on VA by rolling thin-film oven test (RTFOT) at 163 °C for 180 min, which is a common way to simulate the aging process of asphalt binders in laboratory [29,30]. Table 1 lists the properties of AA and VA. Recycled asphalt (RA) was prepared by mixing aged asphalt with BWR together at 160 °C for 1 h, and the content of BWR in aged asphalt ranged from 3.5% to 11.5% with an interval of 2%. “5.5% BWR” in this study indicates that the mass ratio of BWR to aged asphalt is 5.5%.

#### 2.1.2. Bio-Based Warm-Mix Rejuvenator

**Figure 1:**
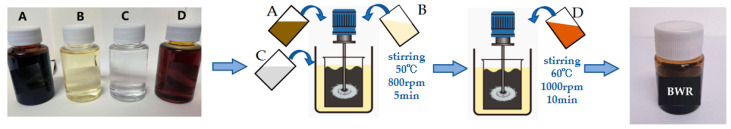
Flowchart of preparing BWR.

The bio-based warm-mix rejuvenator (BWR) is composed of 50.1% waste plant oil (A), 9.8% epoxy triglyceride ester (B), 11.2% naphthenic oil filled with waste rubber (C), and 29.9% oleic acid diethanolamide (D) by mass [23]. Waste plant oil is a mixture of waste rapeseed oil and waste soybean oil, which contains a lot of light components such as saturates and aromatics. Epoxy triglyceride ester is a plasticizer that can improve the low-temperature deformation ability of aged asphalt [31]. Naphthenic oil filled with waste rubber can reduce the viscosity of aged asphalt and provide some light components. Oleic acid diethanolamide is a non-ionic surfactant that can reduce the mixing temperature by changing the surface energy between asphalt and aggregate. Table 2 shows the properties of BWR, and the procedure of preparing BWR is shown in Figure 1.

### 2.2. Experimental Methods

#### 2.2.1. Design and Preparation of Asphalt Blending Samples

**Figure 2:**
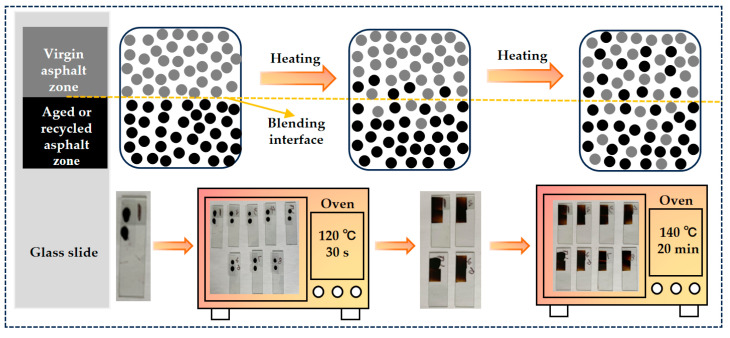
Schematic diagram of design and preparation of asphalt blending samples.

The design and preparation procedure of the asphalt blending samples are described in Figure 2. Due to the blending behavior between virgin asphalt and aged asphalt (or recycled asphalt) being tested by atomic force microscope (AFM), which requires a smooth sample surface, the asphalt blending samples were prepared by the thermal coating method [32]. Firstly, about 0.1 g of hot liquid asphalt binders were poured onto the designated spot of glass slide with a cover glass on and placed in an oven at 120 °C for 30 s to flow naturally to the entire cover glass so that the asphalt binders could be contacted at the blending interface. Then, the asphalt blending samples were taken out horizontally to remove the cover glass and cooled to room temperature. Finally, to investigate the blending phenomenon, the asphalt blending samples were placed in an oven at various blending durations (20 min, 30 min, 40 min) and blending temperatures (120 °C, 140 °C, 160 °C), and the asphalt blending samples at 140 °C for 20 min are used as control groups.

#### 2.2.2. Atomic Force Microscope (AFM) Test

**Figure 3:**
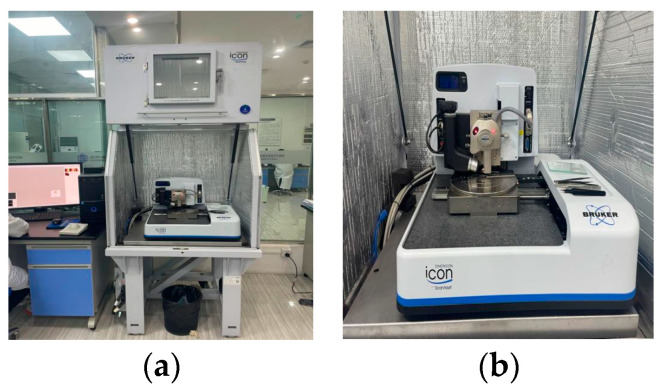
Brook Dimension Icon AFM: (**a**) test instrument; (**b**) test process of tapping mode.

The 2D and 3D micro-morphology of virgin asphalt, aged asphalt, and recycled asphalt were characterized by tapping mode of Brook Dimension Icon AFM, which is shown in Figure 3. The scanning area of 30 μm × 30 μm (512 pixels × 512 pixels) is scanned at a rate of 1.5 Hz. The silicon probe used in AFM test is 3.7 μm in height, 160 μm in length, 40 μm in width, and the elastic coefficient and surface energy are 26 N/m and 1649 mJ/m^2^, respectively.

**Figure 4:**
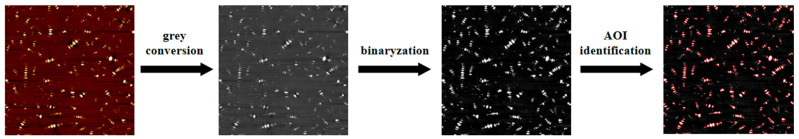
Image processing flowchart of bee-like structures extraction.

Based on the AFM tests, previous studies have found there are obvious bee-like structures in the 2D micro-morphology image of asphalt binders, which exhibit high and low peak-valley structures in 3D micro-morphology image [33]. In this study, several micro-morphology parameters were selected to characterize the variation of asphalt binders after blending. 2D micro-morphology parameters such as the maximum area (*S_max_*), average area (*S_mean_*), total area (*S_total_*), and number (*N*) of bee-like structures were extracted and counted using digital image analysis software (Image-Pro Plus 6.0), and the image processing flowchart is shown in Figure 4. 3D micro-morphology parameters such as the average roughness (*S_a_*), the root mean roughness (*S_q_*), interfacial area ratio (*S_dr_*), and surface material volume (*S_V_*) automatically obtained using Nanoscope analysis software 1.7 were selected to characterize the 3D micro-morphology of asphalt binders. These roughness parameters are defined as follows:

(1) $$S_{a}=\frac{1}{N}\sum_{i}^{N}\left|Z_{i}\right|$$

(2) $$S_{q}=\sqrt{\frac{\sum_{i}^{N}{\left(Z_{i}\right)}^{2}}{N}}$$

(3) $$S_{dr}=\frac{1}{A}\left\{\iint_{A}\left[\sqrt{\left[1+(\frac{\partial z(x,y)}{\partial x}{)}^{2}+(\frac{\partial z(x,y)}{\partial y}{)}^{2}\right]}-1\right]dxdy\right\}$$

(4) $$S_{V}=V_{P}+V_{V}$$

where *Z_i_* is the height of a point over the entire micro-morphology, nm; *N* is the number of peaks and valleys of the 3D micro-morphology; *A* is the surface area of the 3D micro-morphology, μm^2^; *V_P_* is the volume of peaks above the zero plane, nm^3^; and *V_V_* is the volume of valleys below the zero plane, nm^3^.

#### 2.2.3. Definition of Regenerative Blending Degree (RBD)

According to the design of asphalt blending samples, when the samples were heated in the oven, the internal molecular motion of asphalt binders was intensified, and then virgin asphalt and aged asphalt (or recycled asphalt) would diffuse into each other through the blending interface. If the blending degree between virgin asphalt and aged asphalt (or recycled asphalt) becomes higher, the micro-morphology properties should present higher homogeneity [34,35]. Therefore, the regenerative blending degree can be defined by the ratio of the 2D or 3D micro-morphology parameters of virgin asphalt zone and aged asphalt zone after blending as shown in Equation (5). It can be known from Equation (5) that the closer the micro-morphology parameters of asphalt binders are, the higher the regenerative blending degree is.

(5) $$RBD=\frac{P_{\min}}{P_{\max}}\times 100\%$$

where *P*_max_ is the maximum value of the micro-morphology parameters between virgin asphalt and aged asphalt (or recycled asphalt) after blending; *P*_min_ is the minimum value of the micro-morphology parameters between virgin asphalt and aged asphalt (or recycled asphalt) after blending.

## 3. Results

### 3.1. Quantitative Analysis of the Regenerative Blending Degree (RBD)

#### 3.1.1. Based on 2D Micro-Morphology Parameters

1. Virgin Asphalt and Aged Asphalt

**Figure 5:**
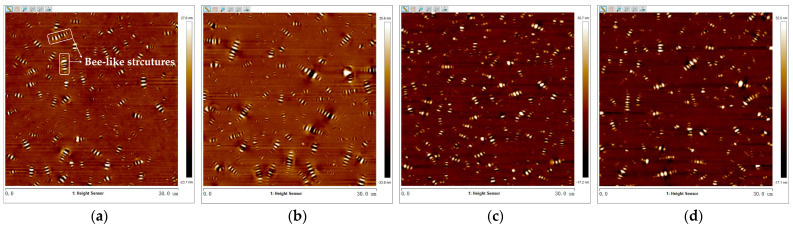
2D micro-morphology images of VA and AA: (**a**) VA before blending; (**b**) AA before blending; (**c**) VA after blending; (**d**) AA after blending.

**Figure 6:**
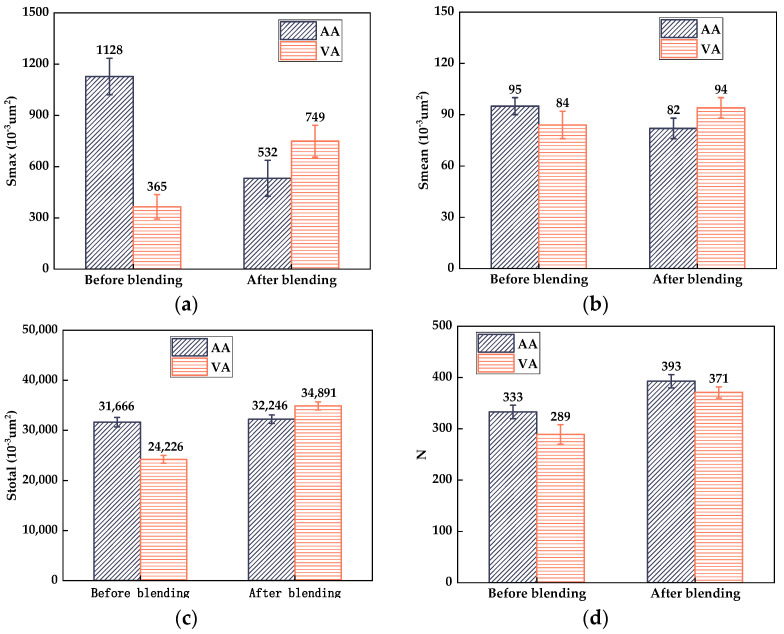
Statistical results of bee-like structures of VA and AA: (**a**) *S_max_*; (b) *S_mean_*; (**c**) *S_total_*; (**d**) *N*.

The 2D micro-morphology images of virgin asphalt and aged asphalt were obtained by tapping mode of the AFM test with the flatten function of Nanoscope analysis software 1.7. The images were presented in Figure 5 where there were many obvious bee-like structures dispersed in asphalt which were attributed to the content of the wax crystals or asphaltenes in asphalt according to the previous studies [32,36]. In comparison with the bee-like structures in Figure 5a,b, the bee-like structures in Figure 5c,d are more uniformly dispersed in size and quantity, which illustrates there was an obvious blending behavior between virgin asphalt and aged asphalt under the action of temperature and duration. To further calculate the regenerative blending degree between virgin asphalt and aged asphalt based on 2D micro-morphology parameters, the maximum area (*S_max_*), average area (*S_mean_*), total area (*S_total_*), and number (*N*) of bee-like structures were quantitatively analyzed by digital image analysis software (Image-Pro Plus 6.0). The statistical results of such parameters of virgin asphalt and aged asphalt before and after blending are shown in Figure 6.

It can be determined from Figure 6 that compared with the 2D micro-morphology parameters between virgin asphalt and aged asphalt before blending, the gap of *S_mean_* value of bee-like structures was almost unchanged, while the gaps of the *S_max_*, *S_total_*, and *N* values were, respectively, reduced from 0.763 μm^2^ to 0.217 μm^2^, 7.44 μm^2^ to 2.645 μm^2^, and 44 to 22 after blending, which indicates there was an obvious blending behavior between virgin asphalt and aged asphalt under a heating temperature and duration [34]. According to Equation (5), the regenerative blending degree (RBD-2D) calculated based on the 2D micro-morphology parameters (*S_max_*, *S_mean_*, *S_total_*, *N*) between virgin asphalt and aged asphalt were 71.1%, 87.2%, 92.4%, and 94.4% at a blending temperature of 140 °C for 20 min, respectively. The average value and standard deviation of RBD-2D were 86.3% and 10.6%.

2. Virgin Asphalt and Recycled Asphalt

**Figure 7:**
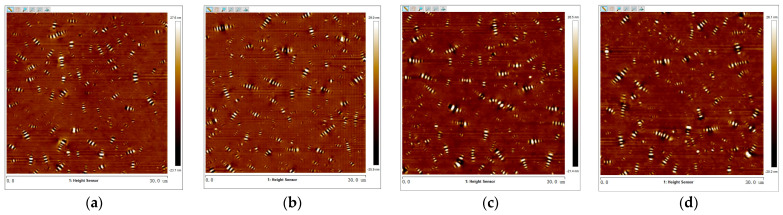
2D micro-morphology images of VA and 3.5% BWR: (**a**) VA before blending; (**b**) 3.5% BWR before blending; (**c**) VA after blending; (**d**) 3.5% BWR after blending.

**Figure 8:**
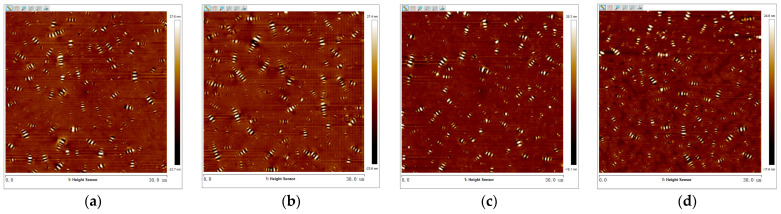
2D micro-morphology images of VA and 5.5% BWR: (**a**) VA before blending; (**b**) 5.5% BWR before blending; (**c**) VA after blending; (**d**) 5.5% BWR after blending.

**Figure 9:**
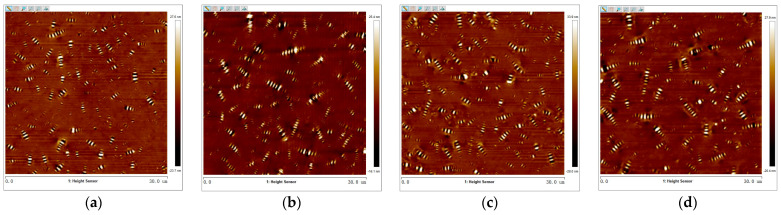
2D micro-morphology images of VA and 7.5% BWR: (**a**) VA before blending; (**b**) 7.5% BWR before blending; (**c**) VA after blending; (**d**) 7.5% BWR after blending.

**Figure 10:**
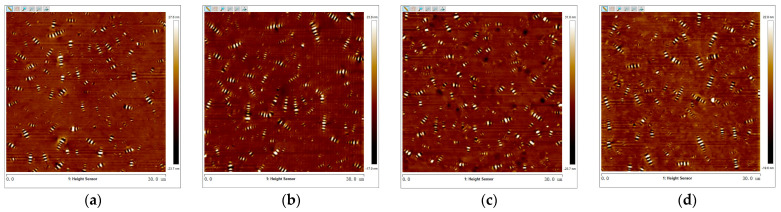
2D micro-morphology images of VA and 9.5% BWR: (**a**) VA before blending; (**b**) 9.5% BWR before blending; (**c**) VA after blending; (**d**) 9.5% BWR after blending.

**Figure 11:**
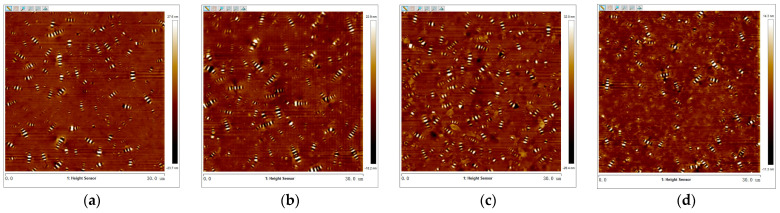
2D micro-morphology images of VA and 11.5% BWR: (**a**) VA before blending; (**b**) 11.5% BWR before blending; (**c**) VA after blending; (**d**) 11.5% BWR after blending.

**Figure 12:**
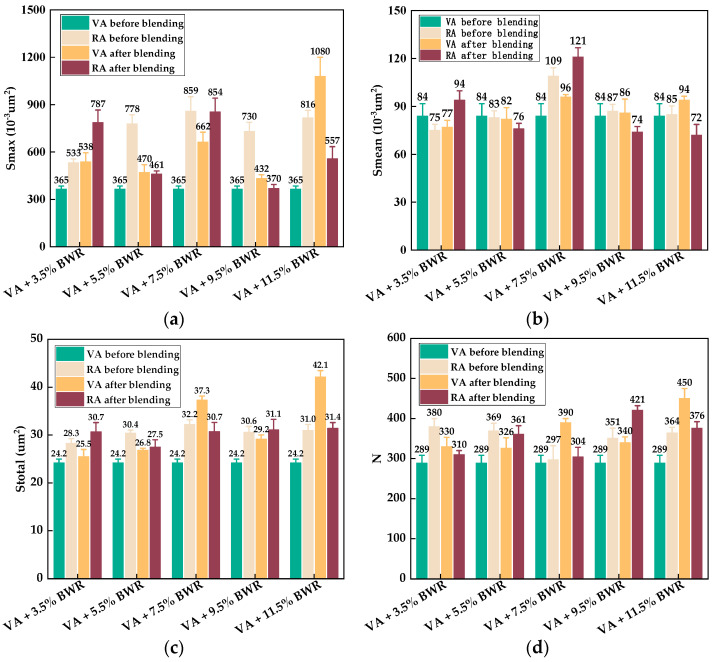
Statistical results of bee-like structures of VA and RA: (**a**) *S_max_*; (**b**) *S_mean_*; (**c**) *S_total_*; (**d**) *N*.

The 2D micro-morphology images between virgin asphalt and various recycled asphalts before and after blending are shown in Figure 7, Figure 8, Figure 9, Figure 10 and Figure 11. It can be seen that there are also many obvious bee-like structures in recycled asphalts which were the same as virgin asphalt and aged asphalt, but compared with the 2D micro-morphology images of virgin asphalt and recycled asphalts before blending, the bee-like structures in 2D micro-morphology images were obviously different after blending at 140 °C for 20 min. These differences in area and quantity of bee-like structures between virgin asphalt and various recycled asphalts were greater than that between virgin asphalt and aged asphalt. Furthermore, with the increase in BWR content in recycled asphalt, this difference seems to be more significant. To accurately analyze the variation of 2D micro-morphology between virgin asphalt and recycled asphalts after blending, the 2D micro-morphology parameters (*S_max_*, *S_mean_*, *S_total_*, *N*) were counted as shown in Figure 12.

It can be found from Figure 12 that the gaps of 2D micro-morphology parameters (*S_max_*, *S_mean_*, *S_total_*) between virgin asphalt and recycled asphalt were smaller than those between virgin asphalt and aged asphalt before blending, which indicates that the bio-based warm-mix rejuvenator (BWR) has an obvious regeneration effect on the microscopic properties of aged asphalt. However, these gaps between virgin asphalt and recycled asphalt were not reduced as much as those between virgin asphalt and aged asphalt after blending, so the regenerative blending degree between virgin asphalt and recycled asphalt could be quite different from that between virgin asphalt and aged asphalt. According to the statistical results of 2D micro-morphology parameters (*S_max_*, *S_mean_*, *S_total_*, *N*) in Figure 12, the regenerative blending degree (RBD-2D) between virgin asphalt and recycled asphalt calculated by Equation (5) is shown in Table 3.

From the results of regenerative blending degree (RBD-2D) calculated in Table 3, at the same blending temperature and duration, the average RBD-2D value between virgin asphalt and 5.5% BWR was the highest at 94.3%, while this value between virgin asphalt and 11.5% BWR was the lowest at 71.4%, and there seems to be no significant relationship between RBD-2D value and the dosage of BWR. The standard deviations of RBD-2D values of virgin asphalt blending with 5.5% BWR, 7.5% BWR, and 9.5% BWR were 3.5%, 2.9%, and 5.2%, which were smaller than those of virgin asphalt blending with 3.5% BWR and 11.5% BWR, which were 10.5% and 13.8%. The reason may be explained by the fact that the viscosity of 5.5% BWR, 7.5% BWR, and 9.5% BWR was closer to virgin asphalt than 3.5% BWR and 11.5% BWR. In addition, compared with *S_max_* value, *S_total_* value, and *N* value, the RBD-2D value calculated by *S_mean_* value is closest to the average value, so it is more appropriate for quantitatively evaluating the blending degree between virgin asphalt and recycled asphalt.

#### 3.1.2. Based on 3D Micro-Morphology Parameters

1. Virgin Asphalt and Aged Asphalt

**Figure 13:**
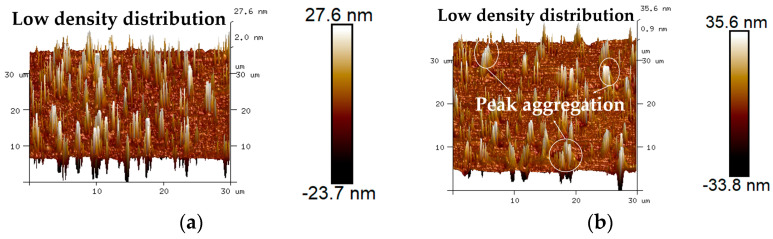

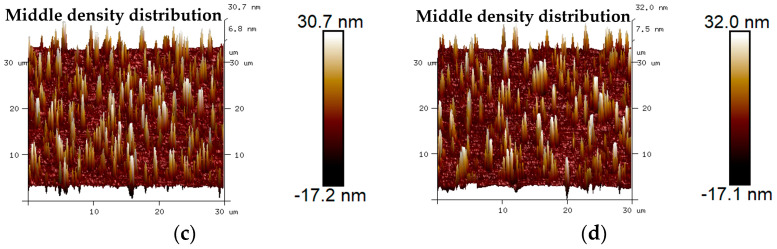
3D micro-morphology images of VA and AA: (**a**) VA before blending; (**b**) AA before blending; (**c**) VA after blending; (**d**) AA after blending.

The 3D micro-morphology of virgin asphalt and aged asphalt before blending is visually shown in Figure 13a,b, and the 3D micro-morphology of such asphalt after blending is shown in Figure 13c,d. These images provide a specific vision for observing the blending phenomenon compared with 2D micro-morphology. The bee-like structures of 2D micro-morphology presented a peak–valley structure with different heights and density distributions in 3D view, and it can be seen in Figure 13 that aged asphalt has a more obvious peak aggregation phenomenon before blending with virgin asphalt, which can be attributed to the increase of asphaltene aggregation in 3D micro-morphology after aging [37]. However, the peak aggregation phenomenon of aged asphalt decreased after blending with virgin asphalt, and the density distributions of the peak–valley structures of virgin asphalt and aged asphalt became more homogeneous, which illustrates that various asphalt binders may have the possibility of fully blending under some conditions.

**Figure 14:**
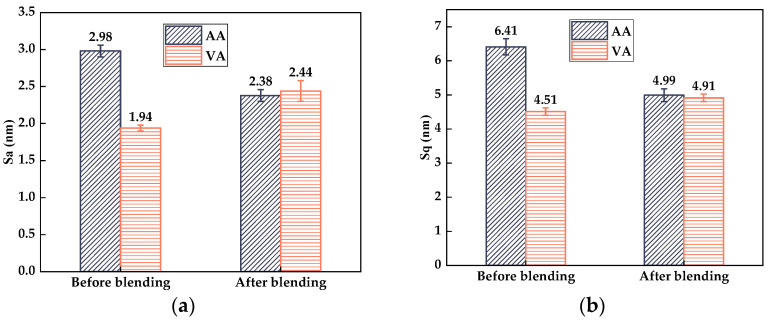

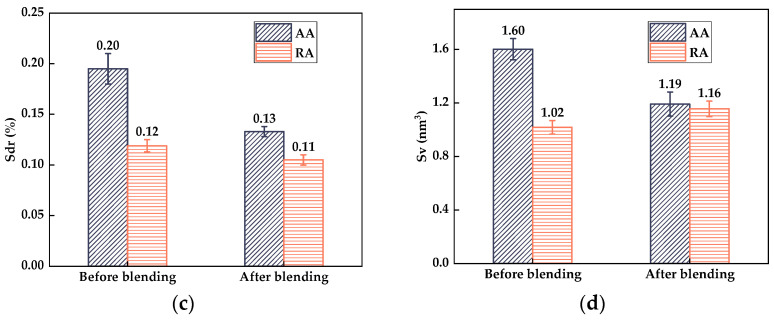
Statistical results of 3D micro-morphology parameters of VA and AA: (**a**) *S_a_*; (**b**) *S_q_*; (**c**) *S_dr_*; (**d**) *S_V_*.

To quantitatively analyze the blending behavior between virgin asphalt and aged asphalt, 3D micro-morphology parameters such as the average roughness (*S_a_*), root mean roughness (*S_q_*), interfacial area ratio (*S_dr_*), and surface material volume (*S_V_*) automatically obtained by Nanoscope analysis software 1.7 were used to characterize the variation in height, surface area, and peak–valley volume of virgin asphalt and aged asphalt during the blending process, and the statistical results of the above parameters are shown in Figure 14. It can be seen intuitively that the gaps of all the 3D micro-morphology parameters between virgin asphalt and aged asphalt become greatly reduced after blending; the gaps of the *S_a_*, *S_q_*, *S_dr_*, and *S_V_* values were reduced from 1.04 nm to 0.06 nm, 1.9 nm to 0.08 nm, 0.08% to 0.02%, and 0.58 nm^3^ to 0.03 nm^3^. According to Equation (5), the regenerative blending degree (RBD-3D) calculated based on 3D micro-morphology parameters (*S_a_*, *S_q_*, *S_dr_*, *S_V_*) between virgin asphalt and aged asphalt was 97.5%, 98.4%, 84.6% and 97.5%, respectively. The average value and standard deviation of RBD-3D were 94.5% and 6.6%. In comparison with the RBD-2D value in Section 3.1.1, the RBD-3D value was larger and its standard deviation was smaller, which was attributed to the fact that the 3D micro-morphology parameters could actually reflect the multi-dimensional information such as the height, surface area, and volume of asphalt binders at nano level, while the RBD-2D value was only calculated by the digital image processing results of the bee-like structures. Thus, the RBD-3D value is more suitable to quantitatively evaluate the blending behavior between virgin asphalt and aged asphalt.

2. Virgin Asphalt and Recycled Asphalt

**Figure 15:**
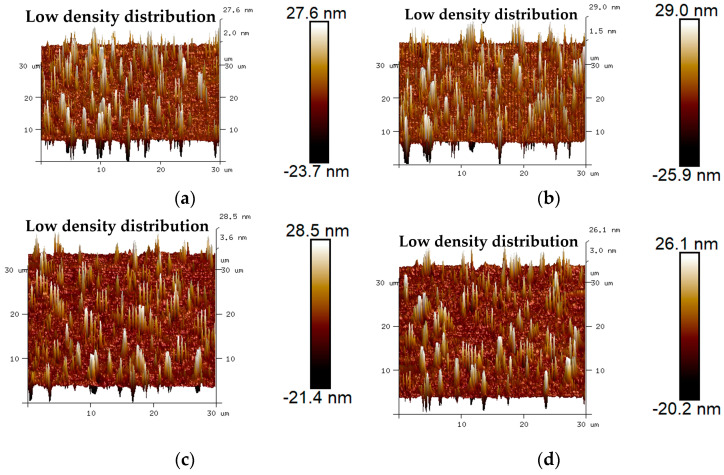
3D micro-morphology images of VA and 3.5% BWR: (**a**) VA before blending; (**b**) 3.5% BWR before blending; (**c**) VA after blending; (**d**) 3.5% BWR after blending.

**Figure 16:**
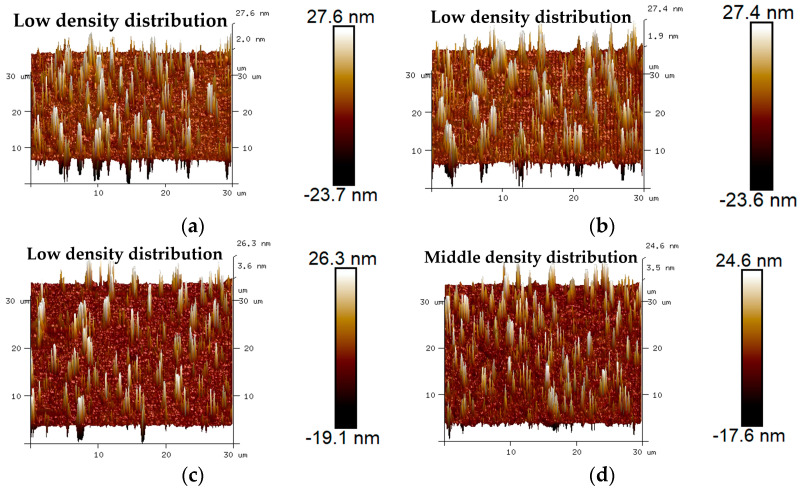
3D micro-morphology images of VA and 5.5% BWR: (**a**) VA before blending; (**b**) 5.5% BWR before blending; (**c**) VA after blending; (**d**) 5.5% BWR after blending.

**Figure 17:**
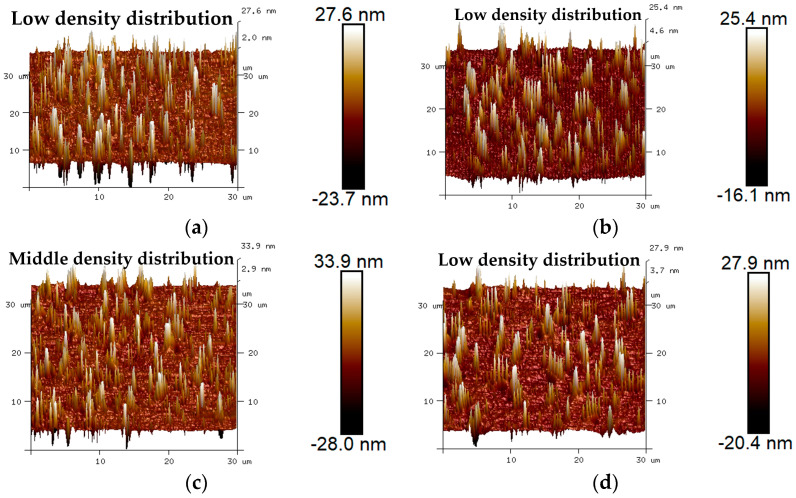
3D micro-morphology images of VA and 7.5% BWR: (**a**) VA before blending; (**b**) 7.5% BWR before blending; (**c**) VA after blending; (**d**) 7.5% BWR after blending.

**Figure 18:**
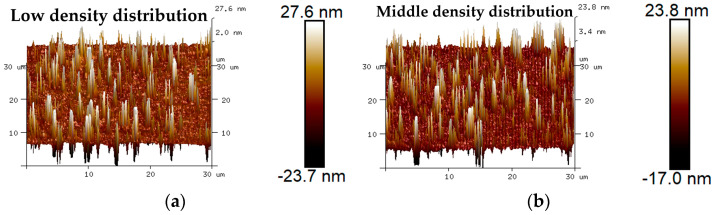

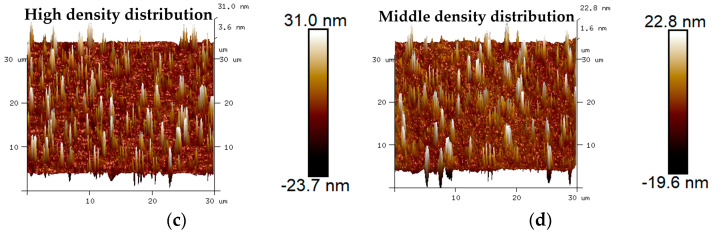
3D micro-morphology images of VA and 9.5% BWR: (**a**) VA before blending; (**b**) 9.5% BWR before blending; (**c**) VA after blending; (**d**) 9.5% BWR after blending.

**Figure 19:**
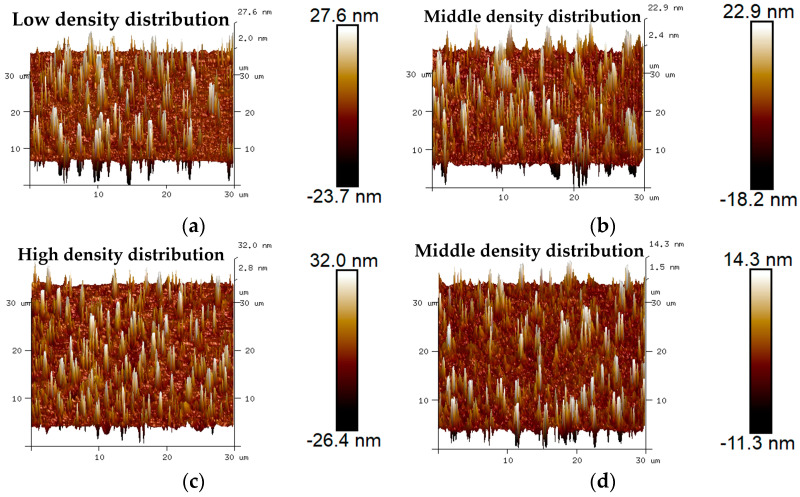
3D micro-morphology images of VA and 11.5% BWR: (**a**) VA before blending; (**b**) 11.5% BWR before blending; (**c**) VA after blending; (**d**) 11.5% BWR after blending.

**Figure 20:**
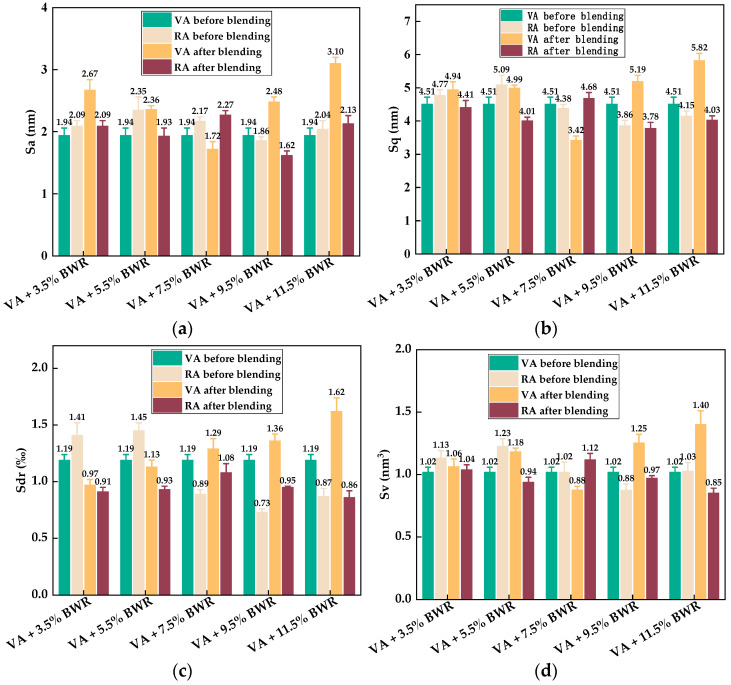
Statistical results of 3D micro-morphology parameters of VA and RA: (**a**) *S_a_*; (**b**) *S_q_*; (**c**) *S_dr_*; (**d**) *S_V_*.

The 3D micro-morphology of virgin asphalt and various recycled asphalts before and after blending are is visually shown in Figure 15, Figure 16, Figure 17, Figure 18 and Figure 19. Compared with aged asphalt in Figure 13b, the peak aggregation phenomenon of recycled asphalt decreased, indicating that adding BWR in aged asphalt has a positive effect on the recovery of its microstructure. It can be recognized that the density distribution of peak-valley structures in 3D micro-morphology of virgin asphalt and recycled asphalts were more different after blending than before, and with the increase of BWR content in recycled asphalts, the density distribution of peak-valley structures in virgin asphalt was more intensive after blending, but they were more dispersed in recycled asphalts. To further analyze the influence of the blending process on the 3D micro-morphology of virgin asphalt and recycled asphalts, the statistical results of each 3D micro-morphology parameter (*S_a_*, *S_q_*, *S_tdr_*, *S_V_*) was shown in Figure 20, and the regenerative blending degree (RBD-3D) between virgin asphalt and recycled asphalts calculated by Equation (5) is shown in Table 4.

It can be seen from Figure 20 that the variation of 3D micro-morphology parameters of virgin asphalt and recycled asphalts was similar to that of 2D micro-morphology parameters during the blending processing. The addition of BWR in recycled asphalts reduced the gaps of 3D micro-morphology parameters between virgin asphalt and recycled asphalts before blending, but these gaps became larger after blending in most cases. Based on the 3D micro-morphology parameters in Figure 20, the regenerative blending degree (RBD-3D) between virgin asphalt and recycled asphalts calculated by Equation (5) is shown in Table 4. The average RBD-3D value gradually decreased with the increase of BWR content in recycled asphalt, and the average RBD-3D value between virgin asphalt and 3.5% BWR was the highest at 89.8%, while this value between virgin asphalt and 11.5% BWR was the lowest at 62.9%. The reason is that the colloidal structures of recycled asphalts are related to the dosage of BWR; the colloidal structures of recycled asphalts and virgin asphalt are more similar and easier to blend with each other when the content of BWR is lower. It can be found that there was an obvious linear correlation between the average RBD-3D value and the dosage of BWR in recycled asphalt, and the correlation coefficient was calculated to be 0.98. Meanwhile, most of the standard deviations of RBD-3D values in Table 4 were smaller than those of RBD-2D values in Table 3, indicating that the discreteness of the data calculated by 3D micro-morphology parameters is lower and more reasonable to evaluate the blending degree between virgin asphalt and recycled asphalts compared with 2D micro-morphology parameters.

### 3.2. Quantitative Analysis of Blending Duration on Regenerative Blending Degree (RBD)

**Figure 21:**
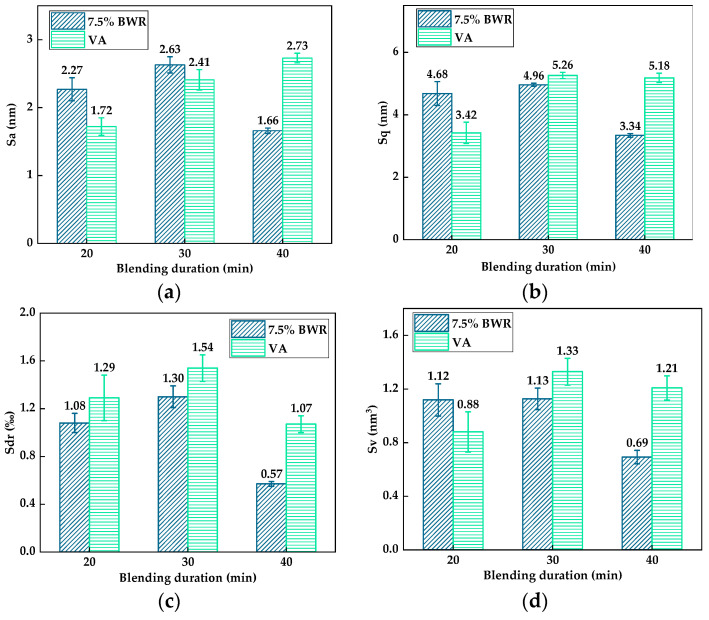
Statistical results of 3D micro-morphology parameters of VA and 7.5% BWR under different durations after blending: (**a**) *S_a_*; (**b**) *S_q_*; (**c**) *S_dr_*; (**d**) *S_V_*.

Previous studies have revealed that the blending duration has an impact on the blending processing between virgin asphalt and aged asphalt [16,25], but the variation of blending degree under different blending conditions after adding bio-based warm-mix rejuvenators in aged asphalt is still unknown. Thus, combined with the above studies of the blending behavior between virgin asphalt and aged asphalt (or recycled asphalts), the 3D micro-morphology parameters were selected to further explore the effect of different blending durations (20 min, 30 min, 40 min) on the blending degree between virgin asphalt and recycled asphalt at 140 °C. The 7.5% BWR was selected as the representative group of recycled asphalt due to its road performance and micro-morphology being the closest to virgin asphalt according to our previous studies [23]. Figure 21 shows the 3D micro-morphology parameters between virgin asphalt and 7.5% BWR under different durations after blending. It can be seen that with an increase in blending duration, the 3D micro-morphology parameters of virgin asphalt and 7.5% BWR presented the same changes, both of which increased first and then decreased. According to Equation (5), the regenerative blending degree (RBD-3D) between virgin asphalt and 7.5% BWR with different blending durations is shown in Table 5.

With the increase in blending duration, the average RBD-3D value between virgin asphalt and 7.5% BWR increased from 77.8% to 88.8% and then sharply decreased to 58.9%, which is over 30% lower than the highest RBD-3D value. The reason can be attributed to the fact that a long blending duration at high temperature would cause the light components of asphalt binder to volatilize and change its colloidal structure, resulting in a decrease in regenerative blending degree between virgin asphalt and recycled asphalt. Therefore, to maintain a better blending state between virgin asphalt and recycled asphalt, the blending duration should be appropriately extended, but not too long. According to the variation of the average RBD-3D values between virgin asphalt and 7.5% BWR under different blending durations, it can be found that there is an optimal blending duration when virgin asphalt is blended with recycled asphalt.

### 3.3. Quantitative Analysis of Blending Temperature on Regenerative Blending Degree (RBD)

**Figure 22:**
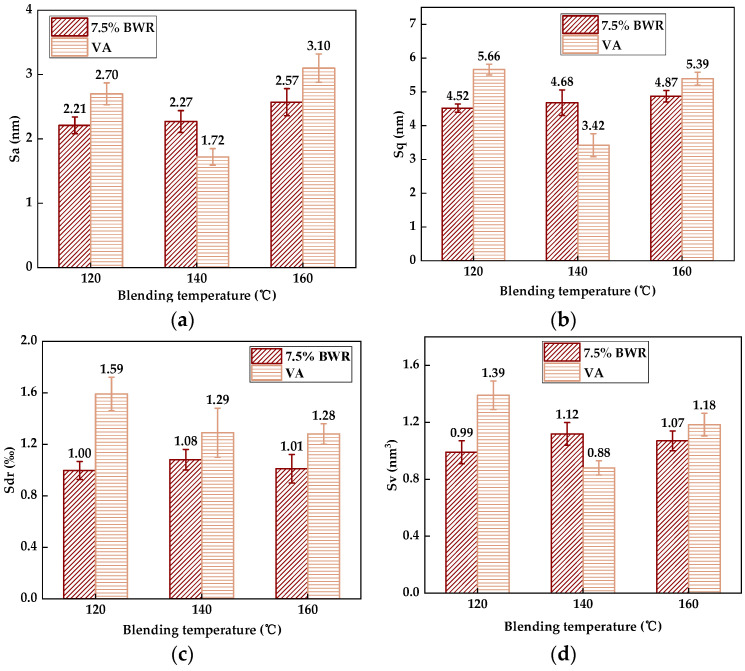
Statistical results of 3D micro-morphology parameters of VA and 7.5% BWR under different blending temperatures: (**a**) *S_a_*; (**b**) *S_q_*; (**c**) *S_dr_*; (**d**) *S_V_*.

Due to asphalt binder being a temperature-sensitive material, its viscosity is closely related to temperature. When the temperature is higher, the asphalt binder will show lower viscosity and higher flow performance [15,38]. Therefore, it is necessary to investigate the quantitative influence of different temperatures on the blending degree between virgin asphalt and recycled asphalt. As with studying the effect of blending duration on blending behavior, Figure 22 shows the 3D micro-morphology parameters between virgin asphalt and 7.5% BWR under different temperatures (120 °C, 140 °C, 160 °C) after blending for 20 min [23]. It can be found that when the temperature was 160 °C, the gaps of 3D micro-morphology parameters were smaller than those at 140 °C and 120 °C, which means the blending processing between virgin asphalt and recycled asphalt is more sufficient. According to Equation (5), the regenerative blending degree (RBD-3D) between virgin asphalt and 7.5% BWR with different blending temperatures is shown in Table 6.

It can be seen from Table 6 that when the blending temperature was 120 °C, 140 °C, and 160 °C, the average RBD-3D values between virgin asphalt and 7.5% BWR were 74.0%, 77.8%, and 85.7%, respectively, indicating that the increase in blending temperature has a positive effect on improving the regenerative blending degree between virgin asphalt and recycled asphalt. This may be because as the blending temperature increased, the viscosity of virgin asphalt and 7.5% BWR gradually approached, and the inter-molecular diffusion movement became easier, leading to a higher average RBD-3D value after blending. Therefore, combined with the above research results of the blending duration on the regenerative degree, it can be known that increasing the blending temperature and prolonging the blending duration appropriately can achieve the best blending effect between virgin asphalt and recycled asphalt.

## 4. Conclusions

In this study, the blending behavior between virgin asphalt and aged asphalt (or recycled asphalt) was quantitatively evaluated by analyzing the regenerative blending degree, which was calculated based on several 2D and 3D micro-morphology parameters obtained by Atomic Force Microscopy (AFM) test. In addition, the effect of various blending durations and temperatures on the regenerative blending degree between virgin asphalt and recycled asphalts was investigated. According to the analysis results, the conclusions are as follows:

(1) In comparison with the average RBD-2D values, the average RBD-3D values were larger and their standard deviations were smaller, which means that the regenerative blending degree calculated by 3D micro-morphology parameters is more accurate for quantitatively evaluating the blending behavior between virgin and aged asphalt (or recycled asphalt);
(2) The average RBD-3D values between virgin asphalt and recycled asphalts gradually decreased with the increase of bio-based warm-mix rejuvenator (BWR) in recycled asphalt, and there was a good linear correlation between the average RBD-3D values and the dosage of BWR in recycled asphalt; the correlation coefficient was calculated to be 0.98;
(3) When the blending duration increased from 20 min to 40 min, the average RBD-3D value between virgin asphalt and 7.5% BWR increased from 77.8% to 88.8% and then decreased to 58.9%, while it continuously increased from 74.0% to 85.7% when the blending temperature increased from 120 °C to 160 °C. Thus, increasing the blending temperature and prolonging the blending duration appropriately can achieve a better blending state between virgin asphalt and recycled asphalt.

This research presented an innovative quantitative method of blending phenomenon between virgin asphalt and aged asphalt by utilizing Atomic Force Microscopy (AFM) incorporating a new bio-based warm-mix rejuvenator (BWR) which was self-developed by the research group, and put forward some practical suggestions to improve the regenerative blending degree between virgin asphalt and recycled asphalts under different blending conditions. In the future, molecular dynamics (MD) simulations can be used to further study the influence of various types of bio-based rejuvenators on the blending degree of virgin asphalt and aged asphalt for theoretical verification.

## Figures and Tables

**Table 1:** Properties of asphalt binders.

Type	Penetration (25 °C, 5 s, 100 g)/0.1 mm	Ductility (5 cm/min, 15 °C)/cm	Softening Point/°C	Viscosity (135 °C)/Pa·s
VA	74	112	48.6	0.375
AA	27.6	5.3	69.1	2.674
3.5% BWR	47.5	23.2	55.9	1.029
5.5% BWR	62.3	69.6	53.4	0.558
7.5% BWR	75.1	102.7	49.7	0.389
9.5% BWR	103.7	119.2	47.4	0.301
11.5% BWR	128.4	128.6	44.9	0.273

**Table 2:** Properties of BWR.

Properties	BWR
Viscosity, mm^2^/s (60 °C)	462
Flash point, °C	245
Viscosity ratio, % (after TFOT, 163 °C)	1.89
Wt change, % (after TFOT, 163 °C)	2.36
Density, g/cm^−3^ (25 °C)	0.89

**Table 3:** Regenerative blending degree (RBD-2D) between virgin asphalt and recycled asphalt.

Type	RBD-2D/%					Standard Deviation/%
	Based on *S_max_* Value	Based on *S_mean_* Value	Based on *S_total_* Value	Based on *N* Value	Average Value	
VA + 3.5% BWR	68.4	81.9	83.9	93.9	82.0	10.5
VA + 5.5% BWR	97.9	92.7	96.4	90.3	94.3	3.5
VA + 7.5% BWR	77.5	79.3	83.8	77.9	79.6	2.9
VA + 9.5% BWR	85.6	86.0	93.5	80.8	86.5	5.2
VA + 11.5% BWR	51.6	76.6	73.8	83.6	71.4	13.8

**Table 4:** Regenerative blending degree (RBD-3D) between virgin asphalt and various recycled asphalts.

Type	RBD-3D/%					Standard Deviation/%
	Based on *S_a_* Value	Based on *S_q_* Value	Based on *S_dr_* Value	Based on *S_V_* Value	Average Value	
VA + 3.5% BWR	78.3	89.3	93.8	98.1	89.9	8.5
VA + 5.5% BWR	81.8	80.4	82.3	79.7	81.1	1.2
VA + 7.5% BWR	75.8	73.1	83.7	78.6	77.8	4.5
VA + 9.5% BWR	65.3	72.8	69.9	77.6	71.4	5.1
VA + 11.5% BWR	68.7	69.2	53.1	60.7	62.9	7.6

**Table 5:** Regenerative blending degree (RBD-3D) between virgin asphalt and 7.5% BWR with different blending durations.

Blending Duration/min	RBD-3D/%					Standard Deviation/%
	Based on *S_a_* Value	Based on *S_q_* Value	Based on *S_dr_* Value	Based on *S_V_* Value	Average Value	
20	75.8	73.1	83.7	78.6	77.8	4.5
30	91.6	94.3	84.4	85.0	88.8	4.9
40	60.8	64.5	53.3	57	58.9	4.8

**Table 6:** Regenerative blending degree (RBD-3D) between virgin asphalt and 7.5% BWR with different blending temperatures.

Blending Temperature/°C	RBD-3D/%					Standard Deviation/%
	Based on *S_a_* Value	Based on *S_q_* Value	Based on *S_dr_* Value	Based on *S_V_* Value	Average Value	
120	81.9	79.9	62.9	71.2	74.0	8.7
140	75.8	73.1	83.7	78.6	77.8	4.5
160	82.9	90.4	78.9	90.7	85.7	5.8

## Data Availability

Data is contained within the article, and no new data were created or analyzed in this study.
